# Umbilical Cord Mercury Concentration as Biomarker of Prenatal Exposure to Methylmercury

**DOI:** 10.1289/ehp.7842

**Published:** 2005-03-31

**Authors:** Philippe Grandjean, Esben Budtz-Jørgensen, Poul J. Jørgensen, Pál Weihe

**Affiliations:** ^1^Institute of Public Health, University of Southern Denmark, Odense, Denmark; ^2^Department of Environmental Health, Harvard School of Public Health, Boston, Massachusetts, USA; ^3^Department of Biostatistics, Institute of Public Health, University of Copenhagen, Copenhagen, Denmark; ^4^Institute of Clinical Research, Odense University Hospital, Odense, Denmark; ^5^Faroese Hospital System, Tórshavn, Faroe Islands

**Keywords:** biomarker, exposure assessment, food contamination, hair analysis, mercury/analysis, methylmercury compounds/analysis, organomercury compounds/blood, pregnancy, prenatal exposure delayed effects, preschool child, seafood, umbilical cord

## Abstract

Biomarkers are often applied to assess prenatal exposure to methylmercury in research and surveillance. In a prospective study in the Faroe Islands, the main exposure biomarkers were the mercury concentrations in cord blood and maternal hair obtained at parturition. We have now supplemented these exposure biomarkers with mercury analyses of umbilical cord tissue from 447 births. In particular, when expressed in relation to the dry weight of the tissue, the cord mercury concentration correlated very well with that in cord blood. Structural equation model analysis showed that these two biomarkers have average total imprecision of about 30%, which is much higher than the laboratory error. The imprecision of the dry-weight–based concentration was lower than that of the wet-weight–based parameter, and it was intermediate between those of the cord blood and the hair biomarkers. In agreement with this finding, regression analyses showed that the dry-weight cord mercury concentration was almost as good a predictor of methylmercury-associated neuropsychologic deficits at 7 years of age as was the cord-blood mercury concentration. Cord mercury analysis can therefore be used as a valid measure of prenatal methylmercury exposure, but appropriate adjustment for the imprecision should be considered.

Exposure assessment is a crucial aspect of environmental epidemiology but remains an inexact science, where validity must be optimized within the confines of efficiency and practicality. Dietary questionnaires constitute a crucial instrument in nutritional epidemiology (Marshall 2003), but they are less useful for food contaminants, because their concentrations usually vary much more than do those of essential nutrients. Instead, environmental epidemiology is relying to an increasing extent on measurements of contaminant concentrations in human tissue samples (Grandjean 1995). Such exposure biomarkers are generally thought to constitute valid measures when laboratory error is carefully controlled. Studies incorporating exposure biomarkers therefore rarely take into account the measurement imprecision.

The ideal exposure biomarker should show a clear-cut relationship to the degree of exposure (Grandjean et al. 1994), but the reality is often that up to several imprecise measures may be available, none of them necessarily an accurate indicator of the true exposure. In regard to methylmercury, substantial information is now available on daily intake levels (European Food Safety Authority 2004), and experimental studies in human volunteers have demonstrated how the dietary intakes may be translated into mercury concentrations in blood (Sherlock et al. 1984) or hair (Hislop et al. 1983). However, these two commonly used exposure biomarkers show only scattered associations (Budtz-Jørgensen et al. 2004), suggesting that their total imprecision significantly exceed routine laboratory errors.

In the first etiologic studies of the so-called Minamata disease, researchers took advantage of the local tradition of saving a dried piece of umbilical cord. Using the cord mercury concentration as an exposure bio-marker, much higher levels were found in patients with Minamata disease compared with control groups (Harada 1977). These retrospective exposure assessments were later extended (Akagi et al. 1998; Dalgård et al. 1994). More recently, mercury was analyzed in a selection of umbilical cords collected from a British birth cohort (Daniels et al. 2004). A sample of umbilical cord is easily collected in connection with births, and the validity of determining mercury as an exposure biomarker therefore deserves to be assessed. However, several factors may affect the characteristics of a cord sample. Vessel contractions within the first couple of minutes after birth (Yao and Lind 1974) will determine the blood content of the cord sample. Umbilical cords differ in thickness and overall appearance, largely due to varying amounts of Wharton’s jelly (Scott and Wilkinson 1978), the amount of which decreases with the duration of gestation (Sloper et al. 1979). The cord mercury concentration is therefore usually expressed in terms of dry weight (Akagi et al. 1998; Dalgård et al. 1994).

The most frequently used sample for methylmercury exposure assessment is scalp hair, especially in field studies (Grandjean et al. 2002). Sampling of hair is noninvasive and painless, and it is a feasible and efficient procedure under most field study conditions. Depending on the rate of hair growth, the mercury concentrations along the hair shaft can represent a calendar of past exposures. Yet environmental mercury vapor may bind to the hair (Yamaguchi et al. 1975), whereas hair permanent treatments can remove much of the endogenous mercury from the hair (Yamamoto and Suzuki 1978; Yasutake et al. 2003). Also, hair color or structure may affect the incorporation of mercury into the hair (Grandjean et al. 2002).

The blood concentration is generally considered the appropriate indicator of the absorbed dose and the amount systemically available. This biomarker is also subject to possible variation. Methylmercury binds to hemoglobin, and the high affinity to fetal hemoglobin results in a higher mercury concentration in cord blood than in maternal blood (Sakamoto et al. 2004). Further, whole-blood mercury concentrations are affected by the hematocrit, and some researchers therefore prefer to measure the mercury concentration in erythrocytes (Sakamoto et al. 2004), although this procedure is more cumbersome. Routine analyses for total mercury concentrations also include inorganic mercury, but cord-blood mercury is almost entirely of the methylated form, for which the placenta does not constitute a barrier (Kelman et al. 1982).

In the absence of a gold standard, statistical correlations can be used to ascertain interrelationships between biomarkers. However, all biomarkers are subject to imprecision, and such data will not provide the validation desired. Factor analysis may be used to determine the total imprecision—the combination of laboratory imprecision and preanalytical variation—of each biomarker (Budtz-Jørgensen et al. 2003). The predictive validity of the biomarkers may also be assessed from their associations with known outcome variables (Grandjean et al. 1999). An extended analysis can be carried out using a structural equation model, where confounders and effect variables are included (Budtz-Jørgensen et al. 2002). Our previous experience using this approach has shown that mercury concentrations in cord blood and in maternal hair are subject to substantial variation, the latter to a greater extent than the former (Budtz-Jørgensen et al. 2004).

The present study was carried out to determine the usefulness of the cord mercury concentration as an exposure biomarker in comparison with more commonly used bio-markers of prenatal methylmercury exposure from maternal seafood consumption. We obtained tissue samples for mercury analysis and relevant information in connection with a prospective birth cohort study initiated in the Faroe Islands (Grandjean et al. 1992). The children were examined in regard to possible developmental neurotoxicity effects at 7 years of age (Grandjean et al. 1997), and the exposure biomarkers could therefore also be compared regarding their predictive validity.

## Materials and Methods

### Cohort formation and sample collection.

A birth cohort of 1,022 subjects was formed from consecutive births between 1 March 1986 and the end of 1987 at the three Faroese hospitals (Grandjean et al. 1992). In connection with each birth, we collected umbilical cord tissue, cord blood, and maternal hair. A questionnaire was administered by the midwife to obtain basic information on the general course of the pregnancy and nutritional habits, including frequencies of dinners based on pilot whale meat or fish, use of alcohol, and tobacco smoking. The study was carried out in accordance with the Helsinki convention and with the approval of the ethical review committee for the Faroe Islands and the institutional review board in the United States.

According to routine obstetric procedures, the cord was clamped 1 min after delivery. Cord blood for mercury analysis was then collected directly from the cord and frozen for later analysis (Grandjean et al. 1992). A 5-cm piece of the cord was cut off with a pair of scissors, stored in a glass vial, and frozen until analysis.

### Cord-tissue analysis.

Upon thawing, the wet weight of the cord tissue sample was determined. No attempt was made to remove any remaining blood. The procedure for mercury analysis has been previously described (Dalgård et al. 1994), but changes in equipment necessitated some adjustments. The specimen was freeze-dried for 48 hr before determination of the dry weight. The heating program for the microwave oven was 10 min at 100% power followed by 5 min at 5% and 10 min at 100% power. The volume of the digested sample used for analysis was 500 μL. The mercury analysis was performed by flow-injection cold-vapor atomic absorption spectrometry (FIMS-400 and AS-90; PerkinElmer, Wellesley, MA, USA). The standard curve was generated by using 0, 2, 4, and 6 μg Hg/L solutions in 4.3 M HNO_3_ (with the addition of 5 mL gold solution, 1 g/L, to 1 L HNO_3_). The analytical method for blood samples was the same, except that freeze-drying and the microwave digestion were omitted. Because umbilical cords from children born in 1986 were used for determination of organochlorine contaminants (Grandjean et al. 2001), many samples were exhausted, and the 447 samples analyzed therefore almost entirely represent the younger cohort children born in 1987 and examined in 1994. Wet weight was not recorded in one analytical series of 25 cords.

In connection with the quality assurance of the cord analyses, tissue-based reference materials with low mercury concentrations were analyzed: BCR 184 (bovine muscle) and BCR 185 (bovine liver; both from IRMM, Geel, Belgium). The total analytical imprecision was estimated to be 20 and 6.3% at mercury concentrations of 0.0045 and 0.0392 μg/g (dry weight), respectively. Given the very low concentrations in these materials, the accuracy was deemed acceptable, with average mercury results of 0.0045 μg/g (certified value, 0.0026 μg/g) and 0.039 μg/g (certified value, 0.044 μg/g), respectively. The cord water content of the cord was mostly about 85–90%, but the total range was 62–95%. In 10 split samples, the wet-weight–based mercury concentration showed an average coefficient of variation (CV) of 17%, whereas concentrations in previously analyzed split freeze-dried samples showed an average CV of 4% (Dalgård et al. 1994), that is, similar to the normal laboratory error.

Other methylmercury exposure biomarkers have been previously described (Grandjean et al. 1992). In addition to full-length hair (~ 9 cm), we also analyzed the proximal 2-cm segment close to the root (Grandjean et al. 2003b). These two approaches represent the exposure during the full pregnancy period and during the third trimester. For some cohort members, one or more specimens were not available, and some hair samples were sufficient only for the full-length analysis.

### Clinical follow-up.

Follow-up of this cohort included an extensive neurobehavioral examination at 7 years of age, where five main outcome tests were selected to represent different brain functions [details provided by Grandjean et al. (1997)]: finger tapping with the preferred hand (motor speed); continued performance test reaction time (attention); Bender Visual Motor Gestalt Test (visuospatial); Boston Naming Test (language); and California Verbal Learning Test—Children Short-term Reproduction (verbal memory). Based on the associations with exposure biomarkers, the main effects were seen in attention and language, with lesser impact on motor speed, verbal memory, and visuospatial performance.

### Statistical analysis.

Following descriptive analyses, logarithmic transformations were used for mercury concentrations that showed skewed distributions, and geometric means were calculated. Interrelationships between the transformed exposure biomarkers were determined by correlation coefficients.

A structural equation model analysis was then carried out using only the exposure biomarkers (Budtz-Jørgensen et al. 2002). In a structural equation model, each of these markers (*M-Hg*) was assumed to be manifestations of the true (unobserved) exposure (*Hg*): log(*M-Hg*) = α_m_ + λ_m_ log(*Hg*) + ɛ_m_. We expressed the true exposure on the scale of the cord-blood concentrations. Thus, the factor loading (λ_m_) is fixed at 1 for this bio-marker, and the intercept (α_m_) is 0. In an additional equation, *Hg* was assumed to depend on the frequency of maternal pilot whale dinners during pregnancy, as indicated by a dietary questionnaire.

In this type of analysis, measurement errors (ɛ_m_) in different markers are usually assumed to be independent. However, we anticipated dependence between error terms in the two hair measurements and between errors in the cord-based measurement. To adjust for such local dependence, we allowed ɛ_m_ for the three cord measures to be associated; likewise, we introduced correlation between the ɛ_m_ terms for the two hair concentrations. We also carried out separate analyses based only on two biomarkers at a time (one based on cord, one on hair) to examine the robustness of the model and to avoid adjustment for local dependence.

In this analysis, standard deviations of error in natural log-transformed variables can be interpreted as error CVs in the untransformed concentrations. In addition, meaningful comparisons of the biomarkers can be obtained from their estimated correlations with the true exposure.

Children with incomplete information on the five exposure variables were included in a missing-data analysis based on the maximum likelihood principle (Little and Rubin 1987). Compared with standard complete case analysis, this approach is more powerful and less likely to yield biased results. Under the usual assumption that the likelihood ratio test statistic follows a chi-squared distribution, the hypothesis of pairs of error terms being of similar size can be tested.

### Outliers identified from scatter plots were excluded in additional analyses.

Using the main outcomes at 7 years of age, we then carried out multiple regression analyses that included the same set of confounders that was originally selected (Grandjean et al. 1997). Instead of the cord-blood mercury concentration (Budtz-Jørgensen et al. 2002; Grandjean et al. 1999), we now used a cord-tissue mercury concentration as the exposure variable. The mercury effect is expressed in terms of the change in the response variable relative to the standard deviation of the response that was associated with a doubling in the mercury concentration (Grandjean et al. 1999).

## Results

**Figure 1:**
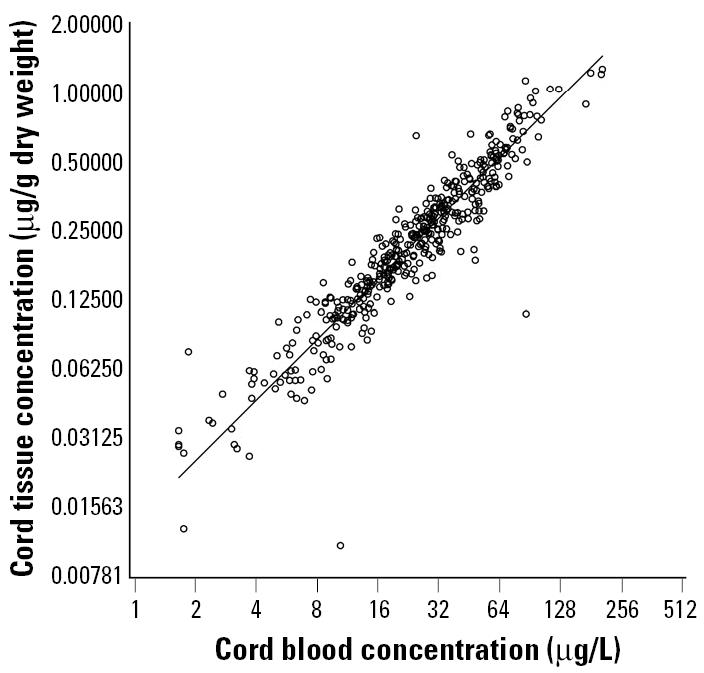
Association between mercury concentrations in cord blood and cord tissue in 447 children from a Faroese birth cohort (*r* = 0.94).

All exposure biomarkers showed wide ranges, where the highest concentration approached 1,000-fold the lowest (Table 1, Figure 1). The medians were very close to the geometric means. The correlations between the bio-markers showed that mercury concentrations in cord tissue and cord blood were closely associated (Figure 1), as were the two hair parameters (Table 2). Overall, the dry-weight cord measurement showed stronger correlations with other mercury biomarkers than did the wet-weight concentration.

The structural equation model provided an excellent fit to the data (*p* = 0.46 for difference between observed and predicted covariances). The cord-blood measurement was the most precise exposure marker, and the dry-weight cord-tissue measure was only slightly inferior, as reflected by the correlations with the true exposure (Table 3). The imprecision of the cord-blood concentration was smaller than that of the other exposure biomarkers (*p* < 0.05). An additional pairwise comparison showed that the dry-weight–based cord-tissue concentration also had a lower imprecision than did the wet-weight parameter (*p* < 0.05). Further analyses were then carried out in submodels including only one cord-based marker and one hair-based marker at a time. The results obtained were very similar to those shown in Table 3, thus supporting the robustness of the model. Likewise, exclusion of outliers changed the results only minimally, although the imprecision of the cord-tissue analysis decreased slightly.

We then performed regression analyses to compare the predictive validity of the exposure biomarkers regarding adverse effects on neurobehavioral development at 7 years of age. The regression coefficients (Table 4) for cord-tissue concentrations generally showed results similar to those previously obtained for cord blood (Grandjean et al. 1999), although some are based on much smaller cohort subgroups with complete data for the cord-tissue biomarkers. For four of five outcome variables, the cord concentration measured in terms of dry weight appeared to be a better predictor than the one expressed in regard to the wet weight.

## Discussion

An imprecise exposure assessment will tend to underestimate the true effect of the exposure and may also complicate confounder adjustment (Carroll 1998). Validation of exposure biomarkers, therefore, is a key to environmental epidemiology studies. However, even superb laboratory repeatability results cannot substantiate the validity of a biomarker in regard to a causative exposure and the associated disease risk. A valid exposure marker must reflect the actual exposure, which is usually unknown.

The present study has employed different statistical strategies to explore this issue. The results show that analysis of cord blood or cord tissue is likely to provide better precision than does maternal hair. Our previous application of structural equation models showed that the imprecision in hair mercury analyses is substantial and can produce underdetermination of neurotoxic impacts of methylmercury exposures (Grandjean et al. 2003a). Other authors have shown a highly scattered association between maternal hair mercury concentrations and subsequent mercury concentrations in the child’s brain obtained at autopsy (Huang et al. 2003). These data are in accordance with the measurement error for the hair mercury parameter found in the present study using a structural equation model. Furthermore, the regression coefficients obtained from using the two cord mercury parameters as exposure variable approximate the results obtained for cord blood (Grandjean et al. 1997, 1999).

Given the large imprecision of the hair mercury parameter and its known variation with hair type and hair color (Grandjean et al. 2002), a better exposure biomarker for prenatal methylmercury is desirable. Cord blood has been recommended as the best available parameter (National Research Council 2000), but sampling of cord blood must consider that coagulation starts soon after clamping of the cord, and clinical circumstances may prevent blood collection in time. The umbilical cord offers advantages because it is easy to sample by noninvasive means, the tissue otherwise being discarded after parturition. The cord is formed mainly during the second and third trimesters, and it reaches two-thirds of its full length by the end of the second trimester (Kaufmann and Scheffen 1998). Assuming a biologic half-life of about 45 days for methyl-mercury (Smith and Farris 1996), the cord mercury concentration is likely to represent a measure of the average mercury burden during the third trimester. It will likely be less sensitive to short-term changes than will the cord-blood mercury concentration.

However, certain caveats must be considered in regard to the variability of cord tissue. The appearance of the umbilical cord varies substantially and is mainly due to differences in water content retained by the gelatinous Wharton’s jelly that surrounds the blood vessels (Scott and Wilkinson 1978; Sloper et al. 1979). The mean water content decreases with increasing duration of gestation, and the fetal end of the cord has a higher water content than does the placental end (Sloper et al. 1979). Because of these considerations, the dry-weight–based mercury concentration would seem to be a more precise parameter than the level expressed on a wet-weight basis. As a contributing factor, the blood content of the cord will depend on the time of clamping, because the cord vessels contract, especially during the first minute after parturition (Yao and Lind 1974).

The analytical reproducibility data document that the dry-weight–based mercury concentration is more precise than the one expressed on a wet-weight basis. Although these laboratory comparisons were based on the intraindividual variability, the interindividual variation in water content is probably greater. In agreement with this finding, the structural equation model shows that the dry-weight cord parameter has a better correlation to the true mercury exposure. Likewise, the predictive validity in regard to neurobehavioral deficits at 7 years of age also favors the dry-weight biomarker.

The findings on biomarker imprecision also need to be considered in light of the literature on methylmercury neurotoxicity. The fact that all exposure biomarkers are much more imprecise than suggested by laboratory quality data suggests that dose–effect relationships may have been underestimated, not just in the Faroes cohort (Grandjean et al. 2003a). Substantial imprecision of an exposure parameter also means that inclusion of confounders in the regression analysis may add to the bias toward the null hypothesis (Budtz-Jørgensen et al. 2003).

Other pollutants in seafood, such as poly-chlorinated biphenyls (PCBs), may also affect the neurobehavioral outcomes (Grandjean et al. 2001) and may also be measured with substantial imprecision. However, structural equation modeling has shown that, even if substantial imprecision is assumed in regard to the Faroese data, PCB exposure does not explain the mercury-associated deficits (Budtz-Jørgensen et al. 2002). Also, as expected for a persistent pollutant such as PCB, this exposure is more closely associated with the hair mercury concentration as a long-term measure of seafood intake, although this marker is clearly inferior to the cord-blood concentration as a marker of methylmercury exposure.

The findings of this study support the use of cord blood as the best available exposure biomarker for methylmercury. Cord tissue is clearly an appropriate alternative, especially when the mercury concentration is measured in relation to the dry weight. Although appropriate for use as an exposure biomarker, adjustment for its imprecision should always be considered.

## Figures and Tables

**Table 1:** Geometric means, 25th–75th percentiles, and total ranges of prenatal methylmercury exposure biomarkers used in a Faroese birth cohort.

Exposure biomarker	No.	Geometric mean	Interquartile range	Total range
Cord blood (μg/L)	996	22.35	13.1–40.4	0.90–351
Cord (μg/g dry weight)	447	0.210	0.132–0.36	0.000–1.28
Cord (μg/g wet weight)	422	0.0249	0.0149–0.044	0.0024–0.23
Full-length hair (μg/g)	1,019	4.17	2.52–7.7	0.17–39.1
Proximal hair (μg/g)	683	4.46	2.76–14.6	0.34–40.5

**Table 2:** Pairwise correlation coefficients for logarithmic transformations of biomarkers of prenatal methylmercury exposure used in a Faroese birth cohort.

	Cord blood	Cord (dry)	Cord (wet)	Hair (full-length)	Hair (proximal)
Cord (dry)	0.940	1			
Cord (wet)	0.907	0.942	1		
Hair (full-length)	0.784	0.732	0.690	1	
Hair (proximal)	0.837	0.781	0.730	0.926	1

**Table 3:** Factor loading (λ), standard deviation of the error term (ɛ), and correlation to the estimated true exposure calculated for five biomarkers of prenatal methylmercury exposure.

Biomarker sample	Factor loading	Error SD	Correlation to true exposure
Cord blood	1	0.29	0.94
Cord (dry)	0.89	0.33	0.91
Cord (wet)	0.87	0.40	0.87
Hair (full-length)	0.84	0.45	0.83
Hair (proximal)	0.88	0.37	0.89

**Table 4:** Numerical change (expressed as percentage of the standard deviation) in five different response variables associated with a doubling in cord-tissue mercury concentrations after adjustment for confounders.

	Cord tissue					
	Dry weight		Wet weight		Cord blood	
Response	No.	β (*p*-value)	No.	β(*p*-value)	No.	β(*p*-value)
Motor speed	411	3.00 (0.47)	388	1.38 (0.74)	820	5.37 (0.05)
Attention	89	29.6 (0.01)	72	27.3 (0.03)	390	15.9 (< 0.0001)
Visuospatial	406	1.70 (0.66)	384	1.63 (0.69)	818	3.83 (0.15)
Language	402	11.3 (0.006)	379	10.1 (0.01)	791	10.5 (< 0.0001)
Verbal memory	392	7.45 (0.08)	370	8.04 (0.07)	797	6.64 (0.019)

For comparison, data for cord blood are also shown (Grandjean et al. 1999). The direction of all effects is toward increasing deficit at higher exposures.
